# Acute diverticulitis: surgery timing in elderly patients

**DOI:** 10.1186/1471-2482-13-S1-A3

**Published:** 2013-09-16

**Authors:** Giovanni Aprea, Antonio Giugliano, Alfonso Canfora, Antonio Ferronetti, Francesco Guida, Melania Battaglini Ciciriello, Antonio Savanelli, Rosario Cuomo, Giovanni Sarnelli, Bruno Amato

**Affiliations:** 1 Department of Gastroenterology, Endocrinology and Surgery, General Surgery Division, University “Federico II” of Naples, Via Pansini, 5, 80131, Naples, Italy

## Background

Diverticular disease is common among the elderly. Its management is complex and offers multiple treatment options.

## Findings

Uncomplicated acute diverticulitis is treated with initial conservative therapy, while cases of recurrence are approached with surgery. Perforated acute diverticulitis (Hinchey I and II) it usually treated with apercutaneous drainage followed by elective colonic resection. Therapeutic failure of percutaneous drainage is an indication for emergency surgery. Acute diverticulitis with peritonitis (Hinchey III and IV) requires emergency surgery. The surgical options are two: one-step or two-step operation. According to the data present in literature one-step operation has a lower mortality and complication rate than the two-step operation approach.

## Conclusions

Acute diverticulitis is a condition that requires surgical management. The timing and the surgical technique depend on the single clinical situation.

## Findings

Diverticular disease is widespread in western countries where the overall prevalence is about 27%. It mostly affects elderly patients, in fact its prevalence increases with age, reaching 65% in the ninth decade [1].

This condition is believed to remain asymptomatic in 80-85% of patients. One fourth of the symptomatic patients (or 5% of all patients) develop diverticulitis, and a small number will develop complicated diverticulitis [2].

Despite the low rate of acute diverticulitis in patients with diverticula, this condition has a significant impact on the resources of general surgery departments [3].

Therefore, the management of diverticular disease must take into account both the clinical condition of the patient and the effects that an inappropriate use of resources can have on the budget of a department.

So patients with a symptomatic diverticulosis require only conservative medical therapy and diet in order to prevent acute episodes, while during episodes ofacute diverticulitis patients require anappropriate medical or surgical therapy.

The aim of this short report is to identify the best therapeutic procedure for acute diverticulitis in elderly patients.

All patients during an episode of uncomplicated diverticulitis should undergo conservative therapy: in mild cases it consists of oral hydration, oral antibiotics (i.e., ciprofloxacin and metronidazol) and antispasmodics, while in moderate and severe cases it’s based on bowel rest, intravenous fluids and intravenous broad-spectrum antibiotics [4].

Currently, surgical resection is not recommended after the first episode, but at least after the second. Indeed,the clinical results, measured in quality-adjusted life-years (QALYs) gained, improve if surgery is performed after the fourth episode [5].

Anyhow elective colectomy should be performed at least three months after the last exacerbation.

The choice of the treatment in case of perforated acute diverticulitis changes according to clinical severity evaluated with the "Hinchey staging system" [4].

CT-guided or US-guided percutaneous drainage may be appropriate for pericolic or distant abscess (Hinchey I and II). Patients should improve within 72 hours. In these cases, because of the high risk of recurrence and sepsis, a subsequent elective colectomy is recommended: it should be performed at least three months from the last acute episode in order to minimize the operative risk.

Patients with purulent and fecal peritonitis (Hinchey III and IV) should undergo emergency colectomy.[4]

Both in elective and in emergency setting, the choice is between two surgical options: Hartmann’s procedure or resection with primary anastomosis with or without a protection stoma. The choice depends on clinical conditions, concomitant diseases and surgeon’s experience.

According to a literature review evaluating the surgical management outcomes in diverticular peritonitis, resection with primary anastomosis should be as safe as Hartmann’s procedure. In fact the mortality rate of the resection with primary anastomosis is less than that of Hartmann’s procedure (9.9% vs. 19.6%). Moreover, also the risk of wound infection is lower in the resection with primary anastomosis compared to Hartmann’s procedure (9.6% vs 29.1%).

Instead the anastomotic leak rate in the resection with primary anastomosis is higher than that of the Hartmann’s procedure (13.9% vs 4.3%).

Nevertheless Hartmann’s procedure has an additional risk due to the presence of the stoma: in fact, the rate of complications of the stomais 10.3% [6].

**Figure 1:**
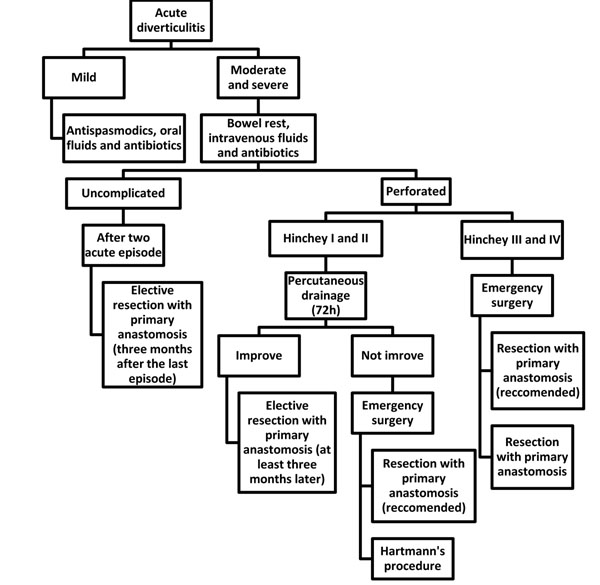
Flow-chart: surgical management of acute diverticulitis.
